# The impact of short-term administration of dapagliflozin on contrast-induced acute kidney injury in patients with type 2 diabetes and renal insufficiency undergoing percutaneous coronary intervention

**DOI:** 10.3389/fmed.2025.1744473

**Published:** 2025-12-15

**Authors:** Shicheng Yang, Shuang Zhu, Xiufeng Zhai, Manxi Liu, Peng Zhang, Naikuan Fu

**Affiliations:** 1 Clinical School of Thoracic, Tianjin Medical University, Tianjin, China; 2 Department of Cardiology, Tianjin University Chest Hospital, Tianjin, China; 3 Tianjin Rehabilitation and Recuperation Center, Joint Logistics Support Force, Tianjin, China; 4 Clinical Medical College of Tianjin Medical University, Tianjin, China

**Keywords:** contrast-induced acute kidney injury, coronary artery disease, dapagliflozin, percutaneous coronary intervention, renal insufficiency, sodium-glucose cotransporter-2 inhibitors, type 2 diabetes

## Abstract

**Background:**

Contrast-induced acute kidney injury (CI-AKI) remains a significant complication in patients with type 2 diabetes mellitus (T2DM) and renal insufficiency undergoing percutaneous coronary intervention (PCI). Many studies have shown that sodium-glucose cotransporter-2 inhibitors (SGLT2i) can improve cardiovascular and renal outcomes in T2DM patients. And chronic administration of SGLT2i has been shown to reduce the risk of CIAKI after PCI in patients with T2DM. However, the impact of short-term SGLT2i administration on the incidence of CIAKI after PCI in T2DM patients with renal insufficiency remains unclear.

**Objective:**

To investigate the impact of short-term (<2 weeks) administration of dapagliflozin on CIAKI in patients with T2DM and renal insufficiency undergoing PCI.

**Methods:**

This retrospective study included patients with T2DM and renal insufficiency who underwent PCI in our hospital, from January to December 2024. The patients were divided into a short-term dapagliflozin group and a control group. Renal function was recorded before PCI, as well as at 48 h and 1 week post-PCI. The primary endpoint was the incidence of CIAKI after PCI in both groups. The secondary endpoints included changes in renal function at 48 h and 1 week post-PCI, as well as the occurrence of major adverse cardiovascular events (MACE) during the 3-month follow-up.

**Results:**

(1) A total of 354 patients with T2DM and renal insufficiency underwent PCI were included in this study, with 183 patients in the short-term dapagliflozin group and 171 patients in the control group. The median duration of short-term dapagliflozin administration before PCI was 3 (2, 6) days, with an average duration of 3.56 ± 1.62 days. (2) The incidence of CIAKI was higher in the short-term dapagliflozin group (14.2%) compared to the control group (7.0%) (*χ^2^* = 4.769, *p* = 0.029). Logistic regression analysis indicated that short-term dapagliflozin administration before PCI was associated with an increased risk of CIAKI (OR = 2.308, 95%CI: 1.002–5.314, *p* = 0.049). (3) During the 3-month follow-up after PCI, Log-rank test showed no significant difference in the incidence of MACE between the two groups (Log-rank *χ^2^* = 0.851, *p* = 0.356). (4) Cox regression analysis revealed that CIAKI significantly affected the short-term prognosis of T2DM patients with renal insufficiency after PCI (HR = 3.025, 95%CI: 1.246–7.343, *p* = 0.014), whereas dapagliflozin did not significantly improve the short-term prognosis of these patients after PCI (HR = 1.024, 95% CI: 0.967–1.084, *p =* 0.415).

**Conclusion:**

Short-term (<2 weeks) dapagliflozin administration may increase the risk of CIAKI in T2DM patients with renal insufficiency undergoing PCI. It is recommended to avoid initiating dapagliflozin in high-risk CIAKI patients prior to PCI.

## 1 Introduction

Percutaneous coronary intervention (PCI) is an effective treatment for coronary artery disease (CAD) that can significantly improve myocardial perfusion, alleviate chest pain symptoms, reduce the risk of adverse cardiovascular events, and enhance both the quality of life and survival rates of CAD patients (1). With the widespread use of PCI, there has been an increase in the use of contrast agents, which has led to a rising incidence of contrast-induced acute kidney injury (CIAKI), particularly among high-risk groups such as those with type 2 diabetes mellitus (T2DM) and renal insufficiency. These patients exhibit a reduced tolerance to contrast agents, which significantly increase their risk of developing CIAKI (2). CIAKI is defined as an acute decline in kidney function occurring within 48–72 h following the administration of contrast agents. Not only does CIAKI prolong hospitalization and increase healthcare costs, but it is also strongly associated with higher rates of cardiovascular events, long-term dialysis dependence, and increased mortality (3).

Dapagliflozin, one of the most widely used sodium-glucose cotransporter-2 inhibitors (SGLT2i) in clinical practice, works by inhibiting the SGLT-2 protein in the proximal renal tubules, which reduces glucose reabsorption and increases urinary glucose excretion. Beyond its glucose-lowering effects, dapagliflozin has been shown to provide significant renal and cardiovascular protection, slowing the progression of chronic kidney disease, reducing urinary protein levels, and improving heart failure outcomes (4–7).

A growing body of evidence suggests that chronic administration of SGLT2i may reduce the risk of CIAKI through mechanisms such as the reversal of renal hyperfiltration, alleviating oxidative stress, and reducing inflammation (8, 9). However, the effects of short-term administration of dapagliflozin—defined as less than 2 weeks—remain inadequately explored. In fact, some studies suggest that short-term SGLT2i therapy could potentially increase the risk of CIAKI due to osmotic natriuresis, dehydration, and renal hemodynamic changes such as vasoconstriction of the afferent arterioles and decreased renal perfusion (10). These changes may be particularly pronounced in high-risk populations, including the elderly and those with multiple comorbidities such as renal insufficiency, heart failure, anemia, or those receiving diuretics. There is currently a lack of consensus regarding the use of SGLT2i in the peri-procedural setting, especially in high-risk patients who are undergoing PCI. Clinical guidelines do not provide clear recommendations on whether dapagliflozin should be withheld or continued in patients at high risk for CIAKI before undergoing PCI. Given the conflicting evidence regarding the effects of short-term dapagliflozin use, it is crucial to clarify whether initiating dapagliflozin in the short term increases the risk of CIAKI in patients with T2DM and renal insufficiency undergoing PCI.

This study aims to investigate the impact of short-term (<2 weeks) dapagliflozin administration on the incidence of CIAKI in T2DM patients with renal insufficiency undergoing PCI. By examining the clinical outcomes in patients who received dapagliflozin for a short duration before PCI, we aim to provide evidence that can inform clinical practice, particularly in high-risk T2DM patients with renal insufficiency, and to evaluate whether short-term dapagliflozin therapy should be avoided in these patients to prevent CIAKI.

## 2 Materials and methods

### 2.1 Study population

**Figure 1:**
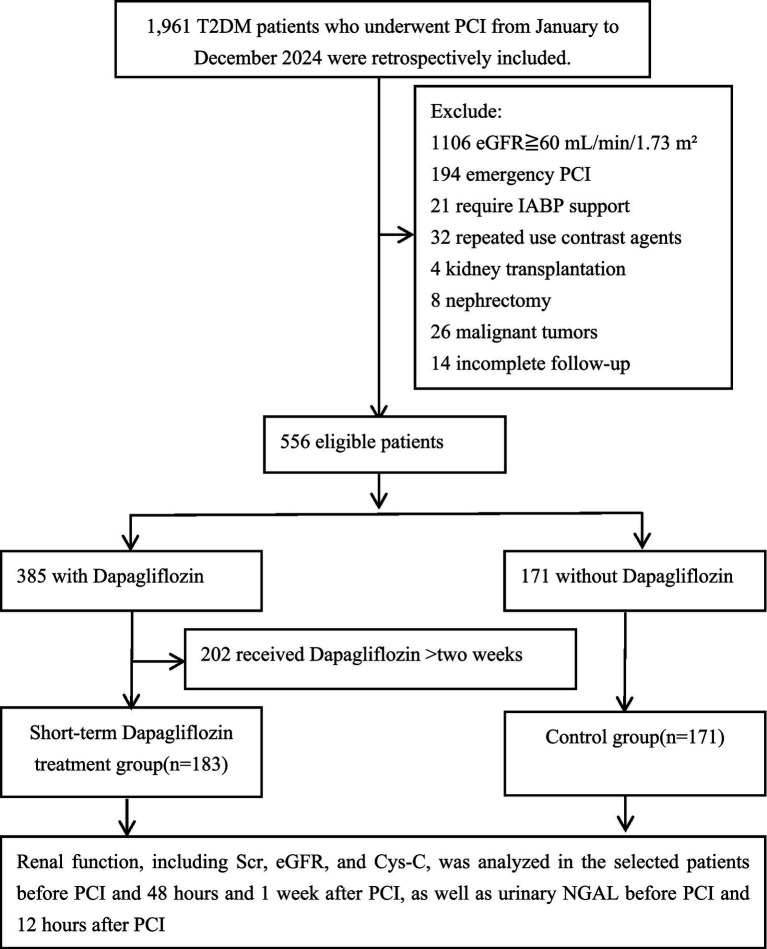
The flowchart of this study. T2DM, type 2 diabetes mellitus; PCI, percutaneous coronary intervention; IABP, intra-aortic balloon pump; eGFR, estimated glomerular filtration rate; Scr, serum creatinine; Cys-C, cystatin C; NGAL, neutrophil gelatinase-associated lipocalin.

We retrospectively included patients with T2DM and renal insufficiency who underwent PCI at the Department of Cardiology, Tianjin University Chest Hospital, from January to December 2024. The inclusion criteria were: (1) Patients either with a long-standing history of T2DM and using hypoglycemic medications, or newly diagnosed individuals who met the diagnostic criteria for T2DM according to the American Diabetes Association guidelines; (2) Patients who fully met the clinical and angiographic indications for PCI, as determined by the interventional cardiologist, based on symptoms and the results of coronary angiography; (3) Patients with an estimated glomerular filtration rate (eGFR) < 60 mL/min/1.73 m^2^, indicating the presence of renal insufficiency; (4) T2DM patients with complete renal function records before and after PCI. The exclusion criteria were: (1) eGFR ≧ 60 mL/min/1.73 m^2^; (2) emergency PCI patients who could not receive sufficient hydration prior to the procedure; (3) patients with cardiogenic shock requiring intra-aortic balloon pump support; (4) patients who had received contrast agents within 2 weeks; (5) patients with malignant tumors; (6) patients with a history of kidney transplantation or nephrectomy; (7) patients allergic to contrast agents or dapagliflozin; (8) patients who had been using dapagliflozin for more than 2 weeks prior to the study; (9) Loss to follow-up during the study period. A flowchart of the study process is shown in Figure 1.

### 2.2 Study protocol

This is a retrospective cohort study. All eligible patients were administered 300 mg of aspirin and 300 mg of clopidogrel orally at least 1 h before PCI to ensure effective platelet inhibition. Following PCI, patients continued with long-term oral therapy consisting of 100 mg of aspirin daily and 75 mg of clopidogrel daily. Data were collected regarding the patients’ baseline medical history, medication history, the initiation time of dapagliflozin, the dose and type of contrast agent used, and the amount of hydration provided. Renal function, including serum creatinine (Scr), eGFR, and cystatin-C (Cys-C), was recorded for the selected patients before PCI and 48 h and 1 week after PCI. Additionally, urinary neutrophil gelatinase-associated lipocalin (NGAL) was recorded for the selected patients before PCI and 12 h after PCI.

The enrolled patients were divided into two groups based on their treatment regimens, a short-term (<2 weeks) dapagliflozin group, patients who received dapagliflozin (AstraZeneca Pharmaceuticals LP, USA, Batch No: H20170119) 10 mg once daily for a period of less than 2 weeks before PCI, and a control group. All enrolled patients received intravenous saline (Shanghai B. Braun Medical Industries Ltd., Batch No. H19993745) 6–12 h before and 6–12 h after PCI at a hydration rate of 1 mL/(kg·h). For patients with heart failure, the hydration rate was reduced to 0.5 mL/(kg·h). The contrast agents used for PCI were either the isotonic contrast agent iodixanol (Yangtze River Pharmaceutical Group Co., Ltd., China, Batch No. H20184002) or the hypotonic contrast agent Iodixolate (Jiangsu Hengrui Medicine Co., Ltd., China, Batch No. H20067896). Patients in the short-term dapagliflozin group received the drug for no more than 2 weeks before PCI. After the procedure, patients continued with long-term dapagliflozin therapy at the same dose of 10 mg daily as part of their routine management for T2DM. All patients were followed up in the outpatient clinic 3 months after discharge, and major adverse cardiac events (MACE) were recorded during hospitalization and follow-up. These included cardiovascular death, recurrent acute myocardial infarction, nonfatal acute stroke, malignant arrhythmias (such as hemodynamically unstable ventricular tachycardia and ventricular fibrillation), and the occurrence of acute heart failure.

The primary outcome of the study was the incidence of CIAKI in the short-term dapagliflozin group compared to the control group. CIAKI was defined as an increase in Scr greater than 26.5 μmol/L above baseline within 48 h after the administration of contrast agents, or an increase greater than 50% above baseline within 1 week (3). The secondary endpoints included changes in renal function at 48 h and 1 week post-PCI, as well as the occurrence of MACE during the 3-month follow-up period after PCI. The eGFR was calculated using the formula: 186 × (Scr/88.4)(μmol/L)^−1.154^ × age (years)^−0.203^ × (0.742) for females. The diagnostic criteria for diabetes were: the presence of diabetic symptoms (polyuria, polydipsia, polyphagia, and weight loss), a postprandial blood glucose level ≧ 11.1 mmol/L, fasting blood glucose ≧ 7.0 mmol/L, or a 2-h post-glucose tolerance test blood glucose ≧ 11.1 mmol/L. The diagnostic criteria for anemia were: hemoglobin levels <120 g/L and red blood cell count <4.5 × 10^12^/L in adult males, and hemoglobin levels <110 g/L and red blood cell count <4.0 × 10^12^/L in adult females. Diuretic use was defined as the administration of furosemide, torsemide (oral or intravenous), or spironolactone during hospitalization. This study was conducted in accordance with the Declaration of Helsinki and was approved by the Medical Ethics Committee of our hospital (approval number: 2023YS-1215-01). All participants signed written informed consent for PCI treatment.

### 2.3 Statistical methods

Statistical analyses were conducted using SPSS 19.0 software (IBM Corp, Armonk, NY, USA). Continuous variables were expressed as mean ± standard deviation (SD) if normally distributed, and median (interquartile range) if non-normally distributed. Differences between groups for continuous variables were evaluated using the independent-samples *t*-test for normally distributed data or the Mann–Whitney *U* test for non-normally distributed data. Categorical variables were presented as frequency (%) and compared using the chi-square test or Fisher’s exact test where appropriate.

For the analysis of renal function and MACE, paired samples *t*-tests were used to compare changes within the groups before and after PCI. Logistic regression analysis was performed to evaluate the risk factors associated with the development of CIAKI, including short-term dapagliflozin administration, baseline renal function, and clinical characteristics. Cox regression analysis was used to assess the influence of CIAKI and dapagliflozin use on short-term prognosis.

The sample size was calculated based on the expected incidence of CIAKI in T2DM patients with renal insufficiency undergoing PCI. Assuming a 10% incidence in the control group and 25% in the dapagliflozin group (9, 10), a total of 298 patients (149 per group) was required to achieve a power of 95% (*α* = 0.05) with a dropout rate of 10%. A two-sided *p*-value of <0.05 was considered statistically significant.

## 3 Results

### 3.1 Comparison of basic characteristics between the two groups

According to the inclusion and exclusion criteria, a total of 354 patients with T2DM and renal insufficiency who underwent PCI from January to December 2024 in our hospital were included in this study. The mean age of the patients was 70.88 ± 6.36 years (range: 42–85 years), with 180 males (50.9%) and 174 females (49.1%). Among them, 183 patients were assigned to the short-term dapagliflozin group, where the median duration of dapagliflozin therapy before PCI was 3 (2, 6) days, with an average duration of 3.56 ± 1.62 days. The control group consisted of 171 patients. The median age in the dapagliflozin group was 72.74 ± 6.14 years, which was slightly higher than that of the control group (69.75 ± 6.53 years, *p* < 0.05). And the left ventricular ejection fraction (LVEF) was lower in the dapagliflozin group (53.74% ± 8.09%) compared to the control group (55.81% ± 8.68%, *p* < 0.05). No significant differences were observed between the two groups for other variables, such as body mass index, diabetes duration, hemoglobin, and history of hypertension, anemia. (*p* > 0.05). See Table 1.

**Table 1:** Comparison of general characteristics between the two groups.

Variables	Dapagliflozin group (183 cases)	Control group (171 cases)	*t*/*z*/*χ*^2^	*p*-value
Age (years)	72.74 ± 6.14	69.75 ± 6.53	4.440	<0.001
Male [*n* (%)]	94 (51.4)	86 (50.3)	0.041	0.840
Body mass index (kg/m^2^)	23.12 ± 1.67	23.06 ± 4.22	0.201	0.352
Smoker [*n* (%)]	71 (38.8)	55 (32.2)	1.697	0.193
drinker[*n* (%)]	52 (28.4)	38 (22.2)	1.788	0.181
Hypertension [*n* (%)]	86 (47.0)	78 (45.6)	0.068	0.795
Systolic blood pressure (mmHg)	136.74 ± 35.52	138.62 ± 36.24	0.493	0.622
Diastolic blood pressure (mmHg)	88.72 ± 20.42	87.64 ± 21.06	0.490	0.625
Acute myocardial infarction [*n* (%)]	66 (36.0)	67 (39.2)	1.632	0.442
Stroke [*n* (%)]	33 (18.0)	28 (16.4)	0.170	0.680
Anemia [*n* (%)]	23 (12.6)	28 (16.4)	1.038	0.308
LVEF (%)	53.74 ± 8.09	55.81 ± 8.68	2.332	0.020
NT-ProBNP (pg/mL)	207.13 (276.77, 652.71)	230.14 (286.44, 684.32)	0.705	0.481
Iodixanol [*n* (%)]	80 (43.7)	64 (37.4)	1.449	0.229
Iodixolate [*n* (%)]	103 (56.3)	107 (62.6)	1.449	0.229
Contrast agent volume (mL)	150.15 ± 40.24	154.68 ± 42.17	1.034	0.302
Hydration volume (mL)	1266.58 ± 382.64	1205.48 ± 378.22	1.510	0.132
Diabetes duration (years)	6.48 ± 2.95	6.06 ± 3.08	1.310	0.191
Fasting glucose (mmol/L)	6.04 ± 1.82	6.19 ± 1.78	0.783	0.434
Postprandial 2-h glucose (mmol/L)	12.68 ± 3.66	12.72 ± 3.75	0.102	0.919
HbA1c (%)	6.88 ± 1.94	6.90 ± 2.01	0.095	0.924
Hemoglobin (g/L)	125.03 ± 16.47	124.54 ± 17.56	0.271	0.489
Triglycerides (mmol/L)	1.65 ± 0.75	1.78 ± 0.82	1.558	0.120
Cholesterol (mmol/L)	3.55 ± 1.02	3.59 ± 1.10	0.355	0.720
HDL-C (mmol/L)	1.07 ± 0.56	1.09 ± 0.48	0.360	0.719
LDL-C (mmol/L)	2.04 ± 0.91	2.11 ± 0.96	0.704	0.483
Statins [*n* (%)]	172 (94.0)	164 (95.9)	0.673	0.412
α-Glucosidase inhibitors [*n* (%)]	82 (44.8)	74 (43.3)	0.084	0.771
Insulin secretagogues [*n* (%)]	66 (36.1)	59 (34.5)	0.094	0.759
Thiazolidinediones [*n* (%)]	31 (16.9)	35 (20.5)	0.725	0.394
GLP-1 RA [*n* (%)]	29 (15.8)	26 (15.2)	0.028	0.868
Insulin [*n* (%)]	64 (35.0)	57 (33.3)	0.106	0.745
Beta-blockers [*n* (%)]	128 (69.9)	116 (67.8)	0.184	0.668
ACEI/ARB [*n* (%)]	115 (62.8)	103 (60.2)	0.254	0.614
Diuretics [*n* (%)]	38 (20.8)	34 (19.9)	0.042	0.387
Calcium antagonists [*n* (%)]	62 (33.9)	65 (38.0)	0.656	0.418

LVEF, left ventricular ejection fraction; HbA1c, glycated hemoglobin; HDL-C, high-density lipoprotein; LDL-C, low-density lipoprotein; GLP-1 RA, glucagon-like peptide-1 receptor agonist; ACEI/ARB, angiotensin-converting enzyme inhibitors/angiotensin II receptor blockers.

### 3.2 Changes in renal function before and after PCI at 48 h and 1 week

There were no statistically significant differences in Scr, eGFR, and Cys-C levels between the two groups before PCI (*p* > 0.05). At 48 h post-PCI, the Cys-C level in the short-term dapagliflozin group was higher compared to the control group, while the eGFR level was lower in the dapagliflozin group compared to the control group, with statistically significant differences (*p* < 0.05). However, at 1 week post-PCI, no significant differences were found in Scr, eGFR, or Cys-C levels between the short-term dapagliflozin group and the control group (*p* > 0.05), as shown in Table 2.

**Table 2:** Changes in renal function before and after PCI at 48 h and 1 week in both groups.

Variables	Dapagliflozin group (*n* = 183)	Control group (*n* = 171)	*t*	*p*-value
Scr (μmol/L)				
PCI pre-treatment	103.71 ± 21.92	102.82 ± 20.67	0.392	0.695
PCI 48 h post-treatment	112.22 ± 26.29^b^	107.12 ± 25.95	2.779	0.067
PCI 1 week post-treatment	106.39 ± 23.64	105.26 ± 22.94	0.456	0.649
eGFR [mL/(min·1.73 m^2^)]				
PCI pre-treatment	52.76 ± 6.48	53.17 ± 6.12	0.611	0.542
PCI 48 h post-treatment	48.45 ± 4.52^a^^,^^b^	50.16 ± 4.64^b^	3.512	0.001
PCI 1 week post-treatment	51.64 ± 5.58	52.77 ± 5.65	1.892	0.059
Cys-C (mg/L)				
PCI Pre-treatment	1.51 ± 0.34	1.50 ± 0.38	0.261	0.794
PCI 48 h post-treatment	2.24 ± 0.66^a^^,^^b^	2.02 ± 0.54^b^	3.419	0.001
PCI 1 week post-treatment	1.55 ± 0.36	1.54 ± 0.39	0.255	0.799

a Compared with the control group (*p* < 0.05).

b Compared with pre-treatment within the same group (*p* < 0.05).

PCI, percutaneous coronary intervention; Scr, serum creatinine; eGFR, estimated glomerular filtration rate; Cys-C, cystatin C.

### 3.3 Changes in urinary NGAL before and 12 h after PCI

There were no statistically significant differences in urinary NGAL level between the two groups before PCI (*p* > 0.05). At 12 h post-PCI, urinary NGAL levels increased in both groups compared to pre-PCI levels, and the short-term dapagliflozin group exhibited higher urinary NGAL level than that in the control group, with a statistically significant difference (*p* < 0.05), as shown in Table 3.

**Table 3:** Changes in urinary NGAL before and 12 h after PCI in both groups.

Variables	*n*	Urinary NGAL (ng/mL)		*t*	*p*
		Pre-treatment	Post-treatment 12 h		
Control group	171	42.78 ± 4.24	50.46 ± 10.52	8.854	<0.001
Dapagliflozin group	183	43.18 ± 4.55	54.22 ± 12.44	11.275	<0.001
*t*		0.854	3.060		
*p*		0.394	0.002		

PCI, percutaneous coronary intervention; NGAL, neutrophil gelatinase-associated lipocalin.

### 3.4 Incidence of CIAKI

A total of 38 patients (10.7%) developed CIAKI during the study period. In the dapagliflozin group, 26 patients (14.2%) developed CIAKI. In the control group, 12 patients (7.0%) developed CIAKI. The difference in the incidence of CIAKI between the two groups was statistically significant (*χ^2^* = 4.769, *p =* 0.029). A comparison of the baseline characteristics between CIAKI and non-CIAKI patients is shown in Table 4. According to the KDIGO staging criteria for acute kidney injury (AKI), among the 38 patients who developed CIAKI in this study, the majority were classified as stage 1 or stage 2, with no patients reaching stage 3 or requiring dialysis. Specifically, 36 patients showed an increase in Scr of more than 26.5 μmol/L above baseline within 48 h after contrast agent administration, or a 50% increase in Scr within 1 week compared to baseline. Only two patients exhibited stage 2, with Scr levels more than doubled but not reaching twice the baseline, and none required dialysis.

**Table 4:** Comparison of general characteristics between CIAKI and non-CIAKI patients.

Variables	CIAKI (*n* = 38)	Non-CIAKI (*n* = 316)	*t/z/χ^2^*	*p*-value
Age (years)	73.16 ± 7.14	70.75 ± 6.63	2.100	0.036
Male [*n* (%)]	19 (50.0)	161 (50.9)	0.012	0.912
Body mass index (kg/m^2^)	22.64 ± 1.56	23.14 ± 3.30	0.921	0.358
Smoker [*n* (%)]	10 (26.3)	116 (36.7)	1.598	0.206
Drinker [*n* (%)]	8 (21.1)	82 (25.9)	0.429	0.512
Hypertension [*n* (%)]	14 (36.8)	150 (47.5)	1.540	0.215
Acute myocardial infarction [*n* (%)]	16 (42.1)	117 (37.0)	0.373	0.541
Stroke [*n* (%)]	6 (15.8)	55 (17.4)	0.062	0.803
Anemia [*n* (%)]	11 (28.9)	40 (12.7)	7.299	0.007
LVEF (%)	52.47 ± 10.21	56.17 ± 8.39	2.506	0.013
eGFR [mL/(min·1.73 m^2^)]	47.43 ± 4.82	51.34 ± 5.24	4.381	<0.001
NT-ProBNP (pg/mL)	644.25 (568.45, 1145.62)	312.62 (325.36, 641.27)	11.212	0.021
Iodixanol [*n* (%)]	16 (42.1)	128 (40.5)	0.036	0.850
Iodixolate [*n* (%)]	22 (57.9)	188 (59.5)	0.036	0.850
Contrast agent volume (mL)	149.22 ± 38.68	155.44 ± 40.26	0.903	0.367
Hydration volume (mL)	1263.16 ± 368.79	1325.56 ± 397.42	0.921	0.358
Diabetes duration (years)	6.72 ± 3.02	6.01 ± 2.56	1.583	0.114
Fasting glucose (mmol/L)	6.33 ± 1.92	6.10 ± 1.70	0.777	0.438
Postprandial 2-h glucose (mmol/L)	13.02 ± 3.94	12.44 ± 3.25	1.015	0.311
HbA1c (%)	7.23 ± 1.84	6.88 ± 2.01	1.023	0.307
Hemoglobin (g/L)	124.53 ± 14.66	124.82 ± 17.26	0.102	0.919
Triglycerides (mmol/L)	1.70 ± 0.68	1.75 ± 0.79	0.374	0.709
Cholesterol (mmol/L)	3.60 ± 0.82	3.73 ± 1.10	0.688	0.492
HDL-C (mmol/L)	1.07 ± 0.44	1.10 ± 0.51	0.347	0.729
LDL-C (mmol/L)	2.06 ± 0.99	1.88 ± 0.89	1.163	0.245
Statins [*n* (%)]	35 (92.1)	301 (95.3)	0.696	0.425
α-Glucosidase inhibitors [*n* (%)]	12 (31.6)	144 (45.6)	2.694	0.101
Insulin secretagogues [*n* (%)]	11 (28.9)	114 (36.1)	0.755	0.385
Thiazolidinediones [*n* (%)]	4 (10.5)	62 (19.6)	1.849	0.174
GLP-1 RA [*n* (%)]	6 (15.8)	49 (15.5)	0.002	0.964
Insulin [*n* (%)]	9 (23.7)	112 (35.4)	2.085	0.149
Beta-blockers [*n* (%)]	30 (78.9)	214 (67.7)	1.996	0.158
ACEI/ARB [*n* (%)]	25 (65.8)	193 (61.1)	0.319	0.572
Diuretics [*n* (%)]	16 (42.1)	56 (17.7)	12.448	<0.001
Calcium antagonists [*n* (%)]	12 (31.6)	115 (36.4)	0.342	0.559
Short-term dapagliflozin use [*n* (%)]	26 (68.4)	157 (49.7)	4.769	0.029

LVEF, left ventricular ejection fraction; HbA1c, glycated Hemoglobin; HDL-C, high-density lipoprotein; LDL-C, low-density lipoprotein; GLP-1 RA, glucagon-like peptide-1 receptor agonist; ACEI/ARB, angiotensin-converting enzyme inhibitors/angiotensin II receptor blockers.

### 3.5 Changes in renal function before PCI, 48 h after PCI, and 1 week after PCI in CIAKI and non-CIAKI patients

In the CIAKI patients, 48 h after PCI, both groups showed a significant increase in Scr and Cys-C levels and a significant decrease in eGFR compared to baseline, with statistical significance (*p*<0.05). In the short-term dapagliflozin group, Scr and Cys-C levels were higher, while eGFR was lower than in the control group, with statistical significance (*p*<0.05). In the non-CIAKI patients, 48 h after PCI, both groups showed an increase in Scr and Cys-C levels and a decrease in eGFR compared to baseline, but the differences were not statistically significant (*p* > 0.05) as shown in Table 5.

**Table 5:** Changes in renal function before PCI and 48 h and 1 week after PCI in CIAKI and non-CIAKI patients.

Variables		CIAKI (*n* = 38)		Non-CIAK (*n* = 316)	
		Dapagliflozin group (*n* = 26)	Control group (*n* = 12)	Dapagliflozin group (*n* = 157)	Control group (*n* = 159)
Scr (μmol/L)	PCI pre-PCI	118.66 ± 30.88	119.41 ± 29.76	100.45 ± 18.77	100.20 ± 18.45
	PCI 48 H post-PCI	159.76 ± 40.08^a^^,^^b^	146.22 ± 36.72^b^	108.36 ± 25.32	104.52 ± 23.82
	PCI 1 week post-PCI	136.42 ± 36.45^b^	128.39 ± 32.52^b^	104.74 ± 21.24	102.69 ± 20.90
eGFR [mL/(min·1.73 m^2^)]	PCI pre-PCI	46.52 ± 7.66	46.84 ± 7.75	54.64 ± 5.33	54.26 ± 5.72
	PCI 48 h post-PCI	36.45 ± 3.62^a^^,^^b^	41.45 ± 4.50^a^^,^^b^	49.75 ± 4.88	52.64 ± 5.65
	PCI 1 week post-PCI	39.64 ± 4.12^b^	42.64 ± 6.89^b^	52.45 ± 5.98	53.64 ± 5.72
Cys-C (mg/L)	PCI pre-PCI	2.01 ± 0.92	1.98 ± 0.90	1.32 ± 0.66	1.30 ± 0.64
	PCI 48 h post-PCI	3.94 ± 1.78^a^^,^^b^	2.68 ± 1.26^a^^,^^b^	2.00 ± 0.96	1.84 ± 0.74
	PCI 1 week post-PCI	3.05 ± 1.34^b^	2.64 ± 1.12^b^	1.49 ± 0.72	1.40 ± 0.56

a Compared with the control group within the same group (*p* < 0.05).

b Compared with pre-treatment within the same group (*p* < 0.05).

PCI, percutaneous coronary intervention; CIAKI, contrast-induced acute kidney injury; Scr, serum creatinine; eGFR, estimated glomerular filtration rate; Cys-C, cystatin C.

### 3.6 Multivariate binary logistic regression analysis

A multivariate binary logistic regression analysis was conducted using the factors that showed statistically significant differences in Table 4 (including diuretic administration, anemia, NT-ProBNP, short-term dapagliflozin administration, LVEF, baseline eGFR, and age) as independent variables, along with factors that may influence the occurrence of CIAKI (including contrast agent dose and hydration volume) as covariates. The results showed that short-term dapagliflozin administration before PCI was associated with an increased risk of CIAKI (OR = 2.308, 95% CI: 1.002–5.314, *p* = 0.049). Additionally, diuretic (OR = 3.681, 95% CI: 1.372–9.879, *p* = 0.010), NT-ProBNP (OR = 2.090, 95% CI: 1.204–3.628, *p* = 0.009), anemia (OR = 3.426, 95% CI: 1.523–7.703, *p* = 0.003), and reduced LVEF (OR = 1.077, 95% CI: 1.006–1.153, *p* = 0.033) were identified as independent risk factors for CIAKI following PCI, as shown in Table 6.

**Table 6:** Risk factors for CIAKI after PCI (multivariable binary logistic regression analysis, *n* = 354).

Variables	*B*	SE	*Waldχ^2^*	*p*	OR	95%CI
Diuretics [*n* (%)]	1.303	0.0504	6.696	0.010	3.681	1.372–9.879
LVEF (%)	0.074	0.035	4.529	0.033	1.077	1.006–1.153
Anemia [*n* (%)]	1.231	0.413	8.869	0.003	3.426	1.523–7.703
Contrast agent volume (mL)	0.170	0.609	0.078	0.780	0.843	0.256–2.783
Hydration volume (mL)	0.000	0.000	0.000	0.996	1.000	0.999–1.001
Age (years)	0.031	0.032	0.953	0.329	1.032	0.969–1.098
NT-ProBNP (pg/mL)	0.737	0.281	6.867	0.009	2.090	1.204–3.628
baseline eGFR [mL/(min·1.73 m^2^)]	0.017	0.013	1.928	0.165	1.018	0.993–1.043
Dapagliflozin [*n* (%)]	0.836	0.4426	3.862	0.049	2.308	1.002–5.314

LVEF, left ventricular ejection fraction; eGFR, estimated glomerular filtration rate; CIAKI, contrast-induced acute kidney injury.

### 3.7 Incidence of MACE

**Figure 2:**
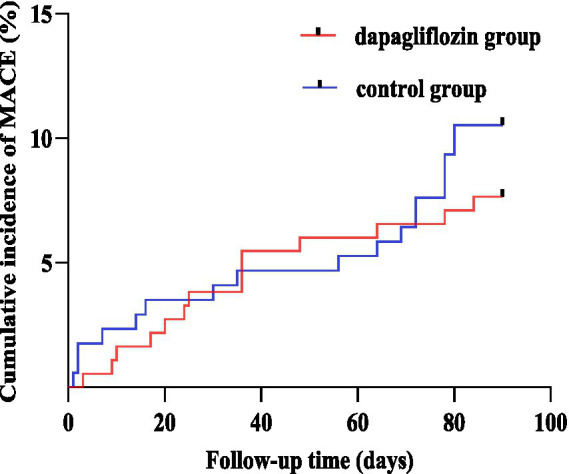
The Kaplan–Meier survival curves for the dapagliflozin group and the control group. MACE, major adverse cardiovascular events.

During the 3-month follow-up after PCI, MACE occurred in both groups. In the short-term dapagliflozin group, there were two cases of recurrent acute myocardial infarction, five cases of repeat revascularization, four cases of acute heart failure, one case of acute stroke, and two cases of malignant arrhythmias. In the control group, there were two cases of recurrent acute myocardial infarction, three cases of repeat revascularization, eight cases of acute heart failure, two cases of stroke, and three cases of malignant arrhythmias. The Log-rank test showed that there was no statistically significant difference in the incidence of MACE between the dapagliflozin group and the control group (Log-rank *χ^2^* = 0.851, *p* = 0.356), as shown in Figure 2.

**Figure 3:**
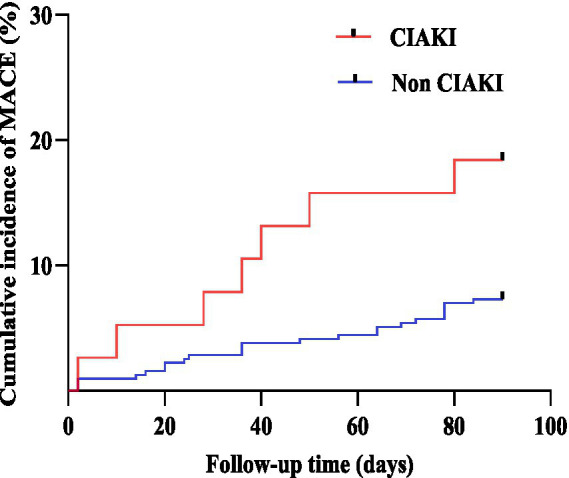
The Kaplan–Meier survival curves for CIAKI and non-CIAKI. MACE, major adverse cardiovascular events; CIAKI, contrast-induced acute kidney injury.

In the CIAKI patients group, there were one case of recurrent acute myocardial infarction, one case of repeat revascularization, one case of stroke, three cases of acute heart failure, and one case of malignant arrhythmias. In the non-CIAKI patients group, there were three cases of recurrent acute myocardial infarction, seven cases of repeat revascularization, nine cases of acute heart failure, two cases of stroke, and four cases of malignant arrhythmias. The Log-rank test revealed that the incidence of MACE was higher in the CIAKI group compared to the non-CIAKI group (Log-rank *χ^2^* = 5.724, *p =* 0.017), as shown in Figure 3.

### 3.8 Multivariate Cox regression analysis

A multivariate Cox regression analysis was performed with the occurrence of MACE as the dependent variable and potential influencing factors, including age, LVEF, baseline eGFR, anemia, NT-ProBNP, CIAKI, and short-term dapagliflozin administration as independent variables. The analysis showed that CIAKI significantly affected the short-term prognosis after PCI in patients with T2DM and renal insufficiency (HR = 3.025, 95% CI: 1.246–7.343, *p* = 0.014). However, dapagliflozin did not significantly improve the short-term prognosis in these patients after PCI (HR = 1.024, 95% CI: 0.967–1.084, *p* = 0.415), as shown in Table 7.

**Table 7:** Influence factors for MACE after PCI (Cox regression analysis, *n* = 354).

Variables	*B*	SE	*Waldχ^2^*	*p*	HR	95%CI
Age (years)	−0.011	0.030	0.123	0.726	0.990	0.933–1.049
Baseline eGFR [mL/(min·1.73 m^2^)]	−0.016	0.022	0.528	0.468	0.984	0.943–1.027
LVEF (%)	0.057	0.028	4.201	0.040	1.059	1.002–1.118
Anemia [*n* (%)]	−0.001	0.012	0.012	0.912	0.999	0.975–1.023
NT-ProBNP (pg/mL)	−0.424	0.318	1.776	0.183	0.654	0.351–1.221
CIAKI [*n* (%)]	1.107	0.452	5.986	0.014	3.025	1.246–7.343
Dapagliflozin [*n* (%)]	0.024	0.029	0.665	0.415	1.024	0.967–1.084

LVEF, left ventricular ejection fraction; eGFR, estimated glomerular filtration rate; CIAKI, contrast-induced acute kidney injury.

## 4 Discussion

In this retrospective cohort study, we investigated the impact of short-term (<2 weeks) dapagliflozin administration on the incidence of CIAKI in patients with T2DM and renal insufficiency undergoing PCI. This study found that the incidence of CIAKI was higher in the short-term dapagliflozin group compared to the control group. Additionally, 12 h post-PCI, urinary NGAL levels were higher in the short-term dapagliflozin group compared to the control group. Our study revealed that short-term dapagliflozin therapy was associated with a higher risk of CIAKI compared to the control group. These findings suggest that, while SGLT2i like dapagliflozin offer long-term renal protection in patients with T2DM and renal insufficiency, their short-term therapy before PCI may pose a higher risk of renal injury, particularly in high-risk populations. Therefore, it is recommended to avoid initiating dapagliflozin in high-risk CIAKI patients before PCI whenever possible.

CIAKI is defined as an increase in Scr by more than 26.5 μmol/L above baseline within 48–72 h after contrast exposure, or an increase exceeding 50% of baseline levels within 1 week. This diagnostic criterion is currently the most widely used in clinical practice (3). However, in the early stages of AKI, Scr has several limitations in clinical application. Scr is influenced by factors such as muscle mass, age, gender, and dietary habits, and its elevation typically requires 24 h to several days after the onset of AKI to reflect acute changes in kidney function, which leads to a delay in the early diagnosis of AKI (11). Cys-C is a small molecular protein produced by nucleated cells, which is filtered by the glomerulus and completely reabsorbed and degraded in the renal tubules. It is not affected by muscle mass, age, or gender, and its levels rise within 6–12 h after the onset of AKI, making it an important marker for early detection of AKI (12). NGAL is a small molecular protein expressed in neutrophils and renal tubular epithelial cells. Under normal conditions, NGAL expression in renal tubular tissue is minimal, but it rises significantly within 3–6 h after the onset of various forms of AKI (13). Therefore, Cys-C and NGAL can serve as sensitive biomarkers for early detection of AKI.

CIAKI is one of the most common iatrogenic causes of AKI. Although the incidence of CIAKI in the general population is relatively low (approximately 1–5%), it can increase to 20–50% in patients with high-risk factors (14). Major risk factors for CIAKI include renal insufficiency (eGFR <60 mL/min/1.73 m^2^), diabetes (especially with concomitant chronic kidney disease), advanced age, heart failure, hypovolemia, anemia, and the use of nephrotoxic drugs. Additionally, the use of large doses or hyperosmolar contrast agents, as well as repeated contrast agent administration within a short period, are important risk factors for CIAKI (15–17). Several studies have shown that CIAKI is closely associated with increased in-hospital mortality, a higher incidence of cardiovascular events, and sustained renal function deterioration (18–20).

Recent studies suggest that contrast agent-induced ischemia and hypoxia in the renal cortex and medulla are key mechanisms in the pathogenesis of CIAKI (21–23). The kidney has a highly complex blood flow distribution, with abundant blood flow in the cortical region and relatively limited blood flow in the medulla, where oxygen tension is naturally lower, putting it in a state of “relative hypoxia” under physiological conditions. This makes the renal medulla more susceptible to further damage from various factors. After exposure to iodinated contrast agents, renal hemodynamics are altered, and the contrast agent can induce vasoconstriction, particularly in the afferent arterioles, leading to reduced renal blood flow, with a significant decrease in medullary perfusion (24). Additionally, contrast agents activate mechanisms such as increased renin release, endothelin-1, and inhibition of nitric oxide and prostaglandin synthesis, which further exacerbate vasoconstriction and reduce medullary blood flow (25). The outer medullary region of the kidney is particularly sensitive to hypoxia, and inadequate perfusion can lead to cellular dysfunction and disturbances in energy metabolism. Moreover, the nephrotoxic effects of contrast agents on renal tubular cells can cause mitochondrial dysfunction, energy metabolism disruption, and cell membrane damage, ultimately leading to renal tubular cell apoptosis or necrosis (26). Furthermore, ischemic and hypoxic conditions activate various oxidative stress pathways, promoting the excessive generation of reactive oxygen species, which causes oxidative stress damage (27), thereby contributing to the development of CIAKI.

SGLT2i have garnered significant attention as a novel class of hypoglycemic agents in recent years, with proven cardiovascular and renal protective effects. Multiple clinical studies have confirmed that dapagliflozin not only effectively delays the decline in renal function but also reduces heart failure hospitalization rates and cardiovascular mortality. Its potential mechanisms include reducing glomerular hyperperfusion and hyperfiltration, delaying renal fibrosis, lowering urinary protein levels, promoting natriuresis, reducing cardiac load, inhibiting myocardial fibrosis, improving ventricular remodeling, lowering blood uric acid levels, reducing body weight, and providing anti-oxidative stress and anti-inflammatory effects (28–30).

Existing studies have shown that chronic administration of SGLT2i has been associated with a reduction in AKI and CIAKI risk (31, 32). Recently, a meta-analysis (33) demonstrated that in populations with long-term time use of SGLT2i (ranging from a minimum of 2 weeks to a maximum of 6 months), and with adequate hydration, SGLT2i reduced the incidence of CIAKI after PCI in patients with T2DM (RR = 0.48, 95% CI: 0.39–0.59, *p <* 0.001). Hua et al. (34) found that dapagliflozin, when used for at least 6 months before contrast exposure, significantly reduced the risk of CIAKI. Paolisso et al. (35) reported that dapagliflozin, with an average use of 7.3 ± 3.0 months before PCI, was an independent predictor of reduced CIAKI risk (OR = 0.356, 95% CI: 0.134–0.943, *p* = 0.038). Nardi et al. (36) found that in heart failure patients who had used SGLT2i for at least 6 months, SGLT2i treatment was associated with a reduced risk of CIAKI (OR = 0.41, 95% CI: 0.16–0.90, *p* = 0.045). Feitosa et al. (37) showed that in T2DM patients who had used SGLT2i for at least 15 days before PCI, SGLT2i did not increase the risk of CIAKI.

Although the cardiovascular and renal protective effects of long-term therapy of SGLT2i in patients with T2DM are widely recognized, the impact of short-term SGLT2i therapy on CIAKI remains unclear. At the same time, clinical data show that short-term SGLT2i therapy, particularly within the first 2 weeks of treatment, can lead to transient increases in Scr and decreases in eGFR. These changes in Scr and eGFR typically occur within 2–4 weeks after treatment initiation (38). This outcome of SGLT2i therapy may increase the risk of AKI in the short term administration, particularly in high-risk populations such as the elderly, those with heart failure, renal insufficiency, anemia, diuretic use, hypovolemia, and contrast agent exposure, where the effect may be more pronounced. In our previous study (39), we retrospectively included T2DM patients who had been taking dapagliflozin for at least 4 weeks prior to PCI (224 patients), with an average duration of use of 10.56 ± 2.62 weeks, and compared them with patients who had not used dapagliflozin (240 patients). We found that the incidence of CIAKI was lower in the dapagliflozin group compared with the control group (5.8% vs. 11.7%, *χ*^2^ = 4.494, *p* = 0.033). Dapagliflozin appeared to reduce the risk of CIAKI (OR = 0.365, 95% CI: 0.176–0.767, *p* = 0.008) (38). However, our previous study did not include patients with severe renal insufficiency, or those with short-term dapagliflozin use before PCI. Therefore, it was not possible to determine whether short-term dapagliflozin use affects CIAKI risk in high-risk patients. For this reason, in the present study, we specifically included renal insufficiency patients to analyze the impact of short-term dapagliflozin use on the risk of CIAKI in this vulnerable population.

In this study, the patients in the short-term (<2 weeks) dapagliflozin group had a median duration of dapagliflozin use before PCI of 3 (2, 6) days, with an average duration of 3.56 ± 1.62 days. These patients were mostly newly initiated on dapagliflozin before PCI. Our results showed that the incidence of CIAKI was higher in the short-term dapagliflozin group compared to the control group. Our logistic regression analysis confirmed that short-term dapagliflozin administration increased the risk of CIAKI (OR = 2.308, 95% CI: 1.002–5.314, *p =* 0.049). Additionally, at 12 h post-PCI, urinary NGAL levels were higher in the short-term dapagliflozin group compared to the control group, suggesting that, during the perioperative period of PCI, initiating dapagliflozin may increase the risk of CIAKI in patients with T2DM and renal insufficiency after PCI. A study on the short-term therapy of SGLT2i and CIAKI incidence reported that patients using SGLT2i for a short duration (1.7 ± 1.4 days) had a significantly higher incidence of CIAKI compared to those using SGLT2i for a long duration (191.5 ± 223.3 days) (20.5% vs. 3.4%, *p* = 0.018) (10). Lopes et al. (40) reported that, in the early stages of dapagliflozin treatment, the co-administration of dapagliflozin with furosemide significantly increased the risk of AKI (*p* = 0.006).

The increased risk of CIAKI associated with short-term therapy of SGLT2i may be related to the following pathophysiological mechanisms: (1) In addition to inhibiting glucose reabsorption, SGLT2i also suppress sodium ion reabsorption in the proximal tubules, leading to an increase in sodium delivery to the macula densa. This negative feedback on the tubuloglomerular feedback system triggers the macula densa to signal granular cells to release renin, thereby activating the renin-angiotensin system. This activation causes vasoconstriction of the afferent arterioles in the kidney, leading to reduced renal blood flow and a decrease in renal perfusion pressure. This effect is particularly pronounced when combined with ACEI or ARBs, as these drugs simultaneously dilate the efferent arterioles, the risk of AKI may be heightened (41). (2) The osmotic natriuresis, diuretic, and blood volume-reducing effects of SGLT2 inhibitors, particularly when used in combination with diuretics, may result in a relative insufficiency of effective blood volume in T2DM patients, thereby increasing the risk of AKI (42). (3) SGLT2i may increase the risk of renal medullary hypoxia, particularly in patients with T2DM. Oxygen tension normally decreases at the corticomedullary junction, reaching levels as low as 30 mmHg in the renal medulla, where blood flow is limited and oxygen tension is inherently low, placing the region in a state of “relative hypoxia” even under physiological conditions. SGLT2i promote increased sodium reabsorption in the distal nephron, which raises oxygen consumption in the renal medulla (43). Moreover, studies have shown that SGLT2i can reduce microvascular renal oxygen tension in the cortex and outer medulla of diabetic rats (44, 45). As the renal medulla in T2DM patients is typically already under conditions of inadequate oxygen supply, SGLT2i may make the renal medulla more susceptible to hypoxia and ischemia, leading to further renal function deterioration. This effect may be more evident in patients with anemia or those receiving contrast agents, thereby increasing the risk of CIAKI.

NGAL as a biomarker for distal tubular injury, Darawshi et al. (46) reported that serum and urinary NGAL significantly increased in hospitalized patients treated with SGLT2i who presented with AKI upon admission, as compared with hospitalized patients treated with SGLT2i who did not develop AKI. Additionally, both serum and urinary NGAL levels were significantly correlated with the degree of acute renal damage. Interestingly, in our study, we also observed a significant increase in urinary NGAL in both groups compared to pre-PCI levels, with the short-term dapagliflozin group showing higher urinary NGAL levels than the control group. These results suggest that short-term dapagliflozin therapy may enhance renal tubular injury following contrast administration by affecting renal medullary hypoxia. Meanwhile, in this study, all patients in the dapagliflozin group continued the treatment throughout the study period without interruption, which avoided the fluctuations in eGFR due to the initiation or withholding of SGLT2i, thus preventing a major confounder of changes in GFR that could influence the study results.

In contrast, chronic therapy of SGLT2i may stimulate erythropoietin production and the expression of renal hypoxia-inducible factors, promoting adaptive responses to ischemia and hypoxia and enhancing tolerance to low oxygen conditions. Furthermore, chronic SGLT2i therapy may improve cardiac function through osmotic diuresis and natriuresis, thereby enhancing renal perfusion, and may reduce the risk of CIAKI through its anti-inflammatory and antioxidative stress effects (9, 47–50).

Therefore, based on the results of this study and the aforementioned research (31–40), the following recommendations are made: (1) For patients who have been receiving long-term dapagliflozin therapy, continuation of the drug before PCI may be safe, as long-term exposure to SGLT2 inhibitors has been shown to improve renal hemodynamics and reduce CIAKI risk. (2) It is advisable to avoid initiating dapagliflozin therapy in high-risk patients immediately before PCI, especially in those with severe renal insufficiency, heart failure, or other comorbid conditions that may predispose them to renal injury. (3) For high-risk patients with CIAKI risk factors, it is recommended to discontinue SGLT2i during the perioperative period of PCI to avoid increasing the risk of CIAKI. (4) According to the clinical guidelines for the use of SGLT2i (51), renal function should be closely monitored post-PCI. For patients with a decrease in eGFR exceeding 30% or those who develop CIAKI, it is recommended to discontinue SGLT2i and promptly address the underlying causes of CIAKI.

## 5 Limitation

Indeed, this study has several limitations. First, it is a single-center, retrospective cohort study with non-randomized grouping, which introduces potential selection bias. In this study, patients in the short-term dapagliflozin group were older than that in the control group and had lower LVEF, this difference may be due to the active use of dapagliflozin by cardiologists for patients with heart failure and T2DM, as heart failure patients are often older. This selection bias may have some impact on the results. Second, the exact mechanism by which short-term dapagliflozin increases the risk of CIAKI in this group cannot be fully explained. A more detailed mechanistic study, would be valuable in further elucidating the physiological and biochemical pathways contributing to the observed renal injury. Third, While our study focused on the impact of short-term dapagliflozin administration (less than 2 weeks), the exact optimal duration of therapy for balancing renal protection and minimizing adverse outcomes remains unclear. Further studies are required to evaluate the optimal time for the initiation of SGLT2i after PCI. Fourth, during PCI treatment, patients are usually anxious and commonly show increased heart rate and blood pressure, leading to hypertension. A small proportion of PCI patients may experience hypotension due to pain or anxiety-induced vasovagal reflexes. A very small number of patients may experience coronary no-reflow or slow-flow phenomena during PCI, leading to hypotension. These blood pressure fluctuations may have some impact on the occurrence of CIAKI.

## 6 Conclusion

Our study provides evidence that short-term dapagliflozin administration may increase the risk of CIAKI in T2DM patients with renal insufficiency undergoing PCI. Clinicians should exercise caution when considering the initiation of dapagliflozin in high-risk patients before PCI. While long-term therapy of SGLT2i has clear benefits, short-term initiation of these drugs may exacerbate renal injury. Careful monitoring of renal function and appropriate timing of dapagliflozin initiation are critical to optimizing patient outcomes and minimizing the risk of CIAKI.

## Data Availability

The raw data supporting the conclusions of this article will be made available by the authors, without undue reservation.
